# A Competitive Multiattribute Group Decision-Making Approach for the Game between Manufacturers

**DOI:** 10.1155/2019/8389035

**Published:** 2019-11-11

**Authors:** Quan Zhang, KeXin Jiang, ManTing Yan, JiYun Ma

**Affiliations:** School of Information Engineering, ShenYang University of Technology, ShenYang, China

## Abstract

Under the competitive market environment, the game between manufacturers comes down to the competitive multiattribute group decision-making problem. In this study, the evaluation information of experts is given in the form of 2-dimension 2-tuple linguistic variables, and an approach is proposed for the competitive multiattribute group decision-making problem based on game theory and evidence theory. Firstly, based on the evidence theory, the attribute values of each situation are obtained by aggregating the 2-dimension 2-tuple linguistic evaluation information given by experts. Secondly, according to the attribute values of every situation, the evidence theory is applied for the second aggregation to obtain the overall values of every situation, and then the game matrix of competitive multiattribute group decision problem is formed. Then, according to the bivariate game matrix, the Nash equilibrium point of competitive multiattribute group decision-making problem is determined based on game theory. Finally, a practical case about the alternative selections for a duopoly problem is used to illustrate the effectiveness and applicability of the proposed approach for the competitive multiattribute group decision-making problem.

## 1. Introduction

With the economic globalization and market competition intensification, the game between manufacturers has become very common, which can be viewed as a multiple attribute group decision-making problem (MAGDM). MAGDM is the process to select the most desirable alternative with respect to multiple conflicting attributes based on the evaluation information given by the invited experts [1]. In a competitive multiattribute group decision-making problem, facing a decision matrix, two decision makers usually have to compete with each other when selecting their most desirable alternative [2]. In [2], a competitive multiattribute decision-making framework was proposed. The alternative selected by one decision maker affects the benefits of the other one. The competing decision makers must search a stable situation to ensure their benefits to be maximum or their loss to be minimum. In the course of competitive multiattribute group decision making, the stable situation is searched with respect to the alternatives by the two competing decision makers, which is called the Nash equilibrium. The evaluation information of every alternative is given by experts to determine the Nash equilibrium.

Because of the complexity of multiple attribute decision-making problems, the evaluation information of every alternative given by experts is always uncertain. In competitive multiattribute group decision-making problems, the uncertain evaluation information from the experts can be expressed in various forms, such as fuzzy linguistic information [3–7] and linguistic term sets [8–12]. Compared with linguistic term sets, the fuzzy linguistic information lacks precision in the final results. But, the original linguistic term sets cannot describe the situation where the evaluation information proposed by experts is between two standard linguistic terms. In order to solve this problem, Herrera and Martínez proposed the concept of 2-tuple linguistics and added the unitary information to the original linguistic variables in order to describe the degree to which the evaluation information deviated from the standard linguistic terms [13]. However, 2-tuple linguistic information does not take into account the degree of uncertainty of evaluation information. For this reason, Zhu et al. [14] proposed the 2-dimension 2-tuple linguistic concept by adding one-dimensional information on the basis of 2-tuple linguistic information to describe the degree of uncertainty of evaluation information. The 2-dimension 2-tuple linguistic information can describe the evaluation information of experts more accurately than original linguistic information.

In order to improve the accuracy of decision-making results as compared with the study in [2], this paper proposes an approach for the competitive multiattribute group decision-making problem of the game between manufacturers, where the experts' evaluation information is in 2-dimension 2-tuple linguistics. To solve the competitive multiattribute group decision-making problem with 2-dimension 2-tuple linguistic evaluation information, it is necessary to aggregate experts' evaluation information. The weighted sum is a common approach to aggregate 2-dimension 2-tuple linguistic evaluation information. However, the evidence theory possesses the ability to describe uncertainty and ignorance. Thus, an approach for aggregating 2-dimension 2-tuple linguistic evaluation information is proposed in this paper based on the evidence theory (e.g., Dempster–Shafer theory). In addition, based on the game matrix of competitive multiattribute group decision problem, the Nash equilibrium point of the problem is determined based on game theory.

The organization of this paper is as follows.  Section 2 reviews the current research on the multiple attribute group decision-making problems and the methods for aggregating 2-dimension 2-tuple linguistic information as well as the applications of evidence theory and game theory in multiple attribute decision-making problems.  Section 3 gives preliminary knowledge about 2-tuple linguistic information, 2-dimension 2-tuple linguistic information, and evidence theory. The competitive multiattribute decision-making problem with two decision makers is described in Section 4.  Section 5 proposes an approach for the competitive multiattribute group decision-making problem with 2-dimension 2-tuple linguistic evaluation information. In Section 6, the application of the proposed approach is illustrated by an example.  Section 7 summarizes the results of this study.

## 2. Literature Review

In order to determine the best solution, in [15], two intuitionistic fuzzy aggregation operators were proposed to aggregate the experts' information and attribute information. Garg and Nancy [16] proposed some new linguistic priority aggregation operators, based on which a multiattribute group decision-making method was proposed too. By considering the priority of attributes and experts at the same time, Verma studied the multiattribute group decision-making problems under the trapezoidal fuzzy linguistic information and proposed a new weighted average priority operator [17].

Xu and Cai [18] established a nonlinear programming model to determine the experts' weights based on the principle that the experts' weights should produce the highest consistency of the experts. On this basis, the authors of [19] pointed out the shortcomings of applying genetic algorithm to solve the nonlinear programming model and proposed a new method to determine the experts' weights by linearizing the nonlinear programming model. The authors of [20] proposed the concept of probabilistic uncertain linguistic term sets and the method for probabilistic uncertain linguistic multiattribute group decision-making problems.

In recent years, many scholars studied the 2-dimension 2-tuple linguistic information. In [21], some power aggregation operators were proposed, including 2-dimension uncertain linguistic power generalized aggregation operator (2DULPGA) and 2-dimension uncertain linguistic power generalized weighted aggregation operator (2DULPGWA), and some properties were discussed, as well as the special cases of them.

The authors of [22] proposed a 2-dimension linguistic lattice implication algebra (2DL-LIA) as a linguistic evaluation set with lattice structure. Motivated by the ideas of dependent aggregation operator, in [23], some 2-dimension linguistic dependent aggregation operators were developed. Yu et al. [24] developed a 2-dimension linguistic weighted averaging (2DLWA) operator and a 2-dimension linguistic ordered weighted averaging (2DLOWA) operator. In [25], some density aggregation operators were proposed based on 2DULVs. Liu and Wang [26] proposed three operators: 2-dimension uncertain linguistic weighted averaging (2DULWA) operator, 2-dimension uncertain linguistic weighted geometric (2DULWG) operator, and 2-dimension uncertain linguistic generalized weighted average (2DULGWA) operator. Wu et al. [27] put forward 2-dimension interval type-2 trapezoidal fuzzy ordered weighted average (2DIT2TFOWA) operator and quasi-2-dimension interval type-2 trapezoidal fuzzy ordered weighted average (quasi-2DIT2TFOWA) operator. However, most of the research on the aggregation methods for 2-dimension linguistic variables as discussed above relied on the weighted average operator. The weighted average operator mainly combines the 2-dimension linguistic information by taking the smallest method. Then, the fusion of the highest-level 2-dimension linguistic information with the lowest-level 2-dimension linguistic information is the lowest level of fusion information, which is very unreasonable. Therefore, the approach proposed in this paper is to use the 2-dimension linguistic information as the reliability of the experts, and the above unreasonable situation will not occur.

Because of the advantages of 2-dimension 2-tuple linguistic information, it is very meaningful to study the competitive multiattribute group decision-making problems with 2-dimension 2-tuple linguistic evaluation information.

Evidence theory was widely used in multiattribute group decision-making problems. The authors of [28] proposed a DS-AHP approach for the multiattribute decision-making problem with incomplete information. The authors of [29] proposed an evidence reasoning approach for the multiattribute decision-making problem with incomplete decision matrix. The authors of [30] applied the Dempster–Shafer theory of evidence to develop a new 2-tuple linguistic representation model for decision making and then put forward some linguistic aggregation operators.

Game theory is widely used in modern management science and produces fruitful research results. Chen and Larbani [31] applied game theory in fuzzy multiattribute decision-making problems, by assuming that fuzzy multiattribute decision making is a game between decision makers and nature. Zhou et al. [32] applied game theory to solve multiattribute decision-making problems and thought that the multiattribute decision-making problem was a game between the attributes. And sometimes, manufacturers are often in a fiercely competitive environment when making decisions. Therefore, this paper proposes a multiattribute group decision-making approach for manufacturers to conduct alternative selections in a competitive environment.

## 3. Preliminaries

Some background knowledge about 2-tuple linguistic information and 2-dimension 2-tuple linguistic information is introduced in this section, as well as evidence theory [13, 14, 33].

### 3.1. 2-Tuple Linguistic Variables

**Definition 1 .**

Let *β* be the result of an aggregation of the indexes of a set of labels assessed in a linguistic term set TS(TS={*S*_0_, *S*_1_,…, *S*_*g*_}), i. e., the result of a symbolic aggregation operation. *β* ∈ [0, *g*], with *g*+1 being the cardinality of TS. Let *i*=round (*β*) and *α*=*β* − *i* be two values such that *i* ∈ [0, *g*] and *α* ∈ [−0.5, 0.5); then, *α* is called symbolic translation [13].

The symbolic translation of a linguistic term *S*_*i*_, is a numerical value assessed in [−0.5, 0.5) that supports the “difference of information” between counting of information *β* ∈ [0, *g*] obtained after a symbolic aggregation operation and the closest value in {0,…, *g*} that indicates the index of the closest linguistic term in TS(*i*=round(*β*)) [13].

A 2-tuple (*S*_*i*_, *α*) is usually used to represent the linguistic information, *S*_*i*_ ∈ TS, and *α* ∈ [−0.5, 0.5), as follows:

 
*S*_*i*_ represents the linguistic label center of the information
 
*α*_*i*_ is a numerical value expressing the value of the translation from the original result *β* to the closest index label, *i*, in the linguistic term set TS(*S*_1_,…, *S*_*g*_), i.e., the symbolic translation [13]

**Definition 2 .**

Let TS={*S*_0_, *S*_1_,…, *S*_*g*_} be a linguistic term set and *β* ∈ [0, *g*] be a value representing the result of a symbolic aggregation operation; then, the 2-tuple that expresses the equivalent information to *β* is obtained with the following function [13]:

(1) $$\begin{array}{c} \Delta :\left[0,g\right]⟶\text{TS}\times \left[-0.5,0.5\right),\\ \Delta \left(\beta \right)=\left(S_{i},\alpha \right),\text{with }\left\{\begin{array}{c} S_{i},i=\text{round }\left(\beta \right),\\ \alpha_{i}=\beta -i,\;\alpha_{i}\in \left[-0.5,0.5\right), \end{array}\right. \end{array}$$

where round(·) is the usual round operation, *S*_*i*_ has the closest index label to “*β*,” and “*α*” is the value of the symbolic translation.

**Definition 3 .**

Let TS={*S*_0_, *S*_1_,…, *S*_*g*_} be a linguistic term set and (*S*_*i*_, *α*_*i*_) be a 2-tuple. There is always a Δ^−1^ function such that from a 2-tuple it returns its equivalent numerical value [13]; *β* ∈ [0, *g*] ⊂ *R*:

(2) $$\begin{array}{c} \Delta^{-1}:\text{TS}\times \left[-0.5,0.5\right)⟶\left[0,g\right]. \end{array}$$

(3) $$\begin{array}{c} \Delta^{-1}\left(S_{i},\alpha_{i}\right)=i+\alpha_{i}=\beta . \end{array}$$

**Definition 4 .**

Let (*S*^(1)^, *α*^(1)^) and (*S*^(2)^, *α*^(2)^) be two linguistic term sets; then, their distance is obtained with the following function [33]:

(4) $$\begin{array}{c} d\left(\left(S^{\left(1\right)},\alpha^{\left(1\right)}\right),\left(S^{\left(2\right)},\alpha^{\left(2\right)}\right)\right)=\left|\Delta^{-1}\left(S^{\left(1\right)},\alpha^{\left(1\right)}\right)-\Delta^{-1}\left(S^{\left(2\right)},\alpha^{\left(2\right)}\right)\right|. \end{array}$$

### 3.2. 2-Dimension 2-Tuple Linguistic Variables

The definition of 2-dimension 2-tuple linguistic variables is given as follows.

**Definition 5 .**

Let TS={*S*_0_, *S*_1_,…, *S*_*g*_1__} be the set of linguistic information evaluation in the first dimension. Let TS^*∗*^={*S*_0_^*∗*^, *S*_1_^*∗*^,…, *S*_*g*_2__^*∗*^} be the set of linguistic information evaluation in the second dimension. Then, TWS=((*S*_*i*_, *α*_*i*_), (*S*_*j*_^*∗*^, *α*_*j*_^*∗*^)) *i*=0,  1,   …,  *g*_1_,  *j*=0,  1,   …,  *g*_2_ is defined as a 2-dimension 2-tuple linguistic term [14].

### 3.3. Evidence Theory

**Definition 6 .**

Let *Ω* be a set of mutually exclusive and collectively exhaustive events indicated by

(5) $$\begin{array}{c} \Omega =\left\{E_{1},E_{2},\ldots ,E_{i},\ldots ,E_{n}\right\}, \end{array}$$

where set *Ω* is called a frame of discernment. The power set of *Ω* is indicated by 2^*Ω*^:

(6) $$\begin{array}{c} 2^{\Omega }=\left\{\phi ,\left\{E_{1}\right\},\ldots ,\left\{E_{n}\right\},\left\{E_{1},E_{2}\right\},\ldots ,\left\{E_{1},E_{2},\ldots ,E_{i}\right\},\ldots ,\Omega \right\}. \end{array}$$

Dempster's rule of combination, denoted by *m*=*m*_1_ ⊕ *m*_2_, is defined as follows [2]:

(7) $$\begin{array}{c} m_{1⊕2⊕\cdots ⊕p}\left(A\right)=\left\{\begin{array}{c} \frac{1}{1-k}\underset{\cap A_{i}=A}{\sum }\overset{p}{\underset{i=1}{\prod }}m_{i}\left(A_{i}\right),\;A\neq \phi ;\\ 0,\;A=\phi ; \end{array}\right. \end{array}$$

with

(8) $$\begin{array}{c} k=\underset{\cap A_{i}=\phi }{\sum }\overset{p}{\underset{i=1}{\prod }}m_{i}\left(A_{i}\right). \end{array}$$

## 4. Problem Descriptions

Let *A* and *B* be two manufacturers involved in the competitive multiattribute group decision problems; then, their collections of alternatives, attributes, and experts are stated as follows.

The set of alternatives for manufacturers *A* and *B* is stated as AS={AS_1_, AS_2_,…, AS_*y*_} and BS={BS_1_, BS_2_,…, BS_*t*_}, respectively.

The set of attributes for manufacturers *A* and *B* is {*C*_1_^(1)^, *C*_2_^(1)^,…, *C*_*n*_^(1)^} and {*C*_1_^(2)^, *C*_2_^(2)^,…, *C*_*e*_^(2)^}, respectively.

The set of experts is {*E*_1_^(1)^, *E*_2_^(1)^,…, *E*_h_^(1)^} and {*E*_1_^(2)^, *E*_2_^(2)^,…, *E*_*s*_^(2)^} for manufacturers *A* and *B*, respectively. In this study, the experts express their evaluation information on the alternatives against every attributes in the form of 2-dimension 2-tuple linguistic variables, according to the different strategies of opponent. The standard linguistic term set of the first dimension is TS={*S*_0_, *S*_1_,…, *S*_*g*_1__}. The standard linguistic term set of the second dimension is TS^*∗*^={*S*_0_^*∗*^, *S*_1_^*∗*^,…, *S*_*g*_2__^*∗*^}. The utility value of the first dimension linguistic term set is *u*(TS)={*u*(*S*_0_), *u*(*S*_1_),…, *u*(*S*_*g*_1__)}. Thus, the competitive multiattribute group decision matrix is obtained as follows:

(9) $$\begin{array}{c} D^{\left(1\right)}={\left(a_{ij}^{\left(l\right)}\;\left|\;\right.\text{BS}\right)}_{y\times n},\\ D^{\left(2\right)}={\left(b_{ij}^{\left(r\right)}\;\left|\;\right.\text{AS}\right)}_{\text{t}\times e}, \end{array}$$

where *a*_*ij*_^(*l*)^=(TL_*ij*1_^(*l*)^, TL_*ij*1_^(*l*)*∗*^)=((*S*_*ij*1*p*_^(*l*)^, *α*_*ij*1_^(*l*)^), (*S*_*ij*1*q*_^(*l*)*∗*^, *α*_*ij*1_^(*l*)*∗*^)),  *i*=1,2,…, *y*,  *l*=1,2,…, *h*,  *j*=1,2,…, *n*,  *p*=0,1,…, *g*_1_,  *q*=0,1,…, *g*_2_ is the evaluation value of the alternative AS_*i*_ under the attribute *C*_*j*_^(1)^ given by expert *E*_l_^(1)^ for different strategies chosen by manufacturer *B* and *b*_*ij*_^(*r*)^=(TL_*ij*2_^(*r*)^, TL_*ij*2_^(*r*)*∗*^)=((*S*_*ij*2*x*_^(*r*)^, *α*_*ij*2_^(*r*)^), (*S*_*ij*2*v*_^(*r*)*∗*^, *α*_*ij*2_^(*r*)*∗*^)), *i*=1,2,…, *t*,  *r*=1,2,…, *s*,  *j*=1,2,…, *e*,  *x*=0,1,…*g*_1_,  *v*=0,1,…, *g*_2_ is the evaluation value of the alternative BS_*i*_ under the attribute *C*_j_^(2)^ given by expert *E*_rz_^(2)^ for different strategies chosen by manufacturer *A*.

**Figure 1:**
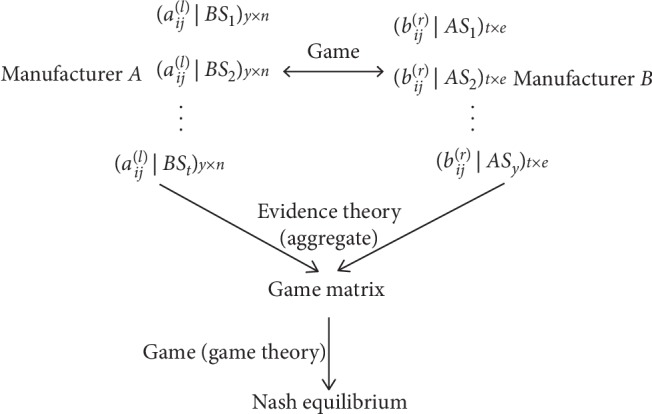
The competitive multiattribute decision framework.

As shown in Figure 1, the problem focused in this study is about the game between manufacturer *A* and manufacturer *B*, where experts of both sides give their evaluation information on the alternatives against the corresponding attributes in the form of 2-dimension 2-tuple linguistic variables. The purpose is to determine the Nash equilibrium for manufacturer *A* and manufacturer *B*.

## 5. An Approach to the Competitive Multiattribute Group Decision-Making Problems with 2-Dimension 2-Tuple Linguistic Variables

In order to solve the competitive multiattribute group decision-making problems with 2-dimension 2-tuple linguistic variables, three stages are employed to obtain the game matrix and determine the Nash equilibrium. In stage one, the evidence theory of Dempster and Shafer is applied and modified to aggregate the experts' evaluation information of 2-dimension 2-tuple linguistic variables. In stage two, the attribute values are integrated to obtain the game matrix. In stage three, according to the obtained game matrix, the Nash equilibrium point for the competitive multiattribute group decision-making problem is determined based on game theory.

### 5.1. Aggregate Experts' Evaluation Information of 2-Dimension 2-Tuple Linguistic Variables Based on Evidence Theory

Based on evidence theory, the following steps are employed to aggregate 2-dimension 2-tuple linguistic information given by experts:

*Step 1*. Calculate the basic probability distribution function
Suppose TL_*ij*1_^(*l*)^=(*S*_*ij*1*p*_^(*l*)^, *α*_*ij*1_^(*l*)^), TL_*ij*1_^(*l*)*∗*^=(*S*_*ij*1*q*_^(*l*)*∗*^, *α*_*ij*1_^(*l*)*∗*^), then the basic probability distribution function is as follows:
  1. When 0 < *α*_*ij*1_^(*l*)^ < 0.5, m_*l*_(*S*_*ij*1*p*_^(*l*)^)=1 − *α*_*ij*1_^(*l*)^, m_*l*_(*S*_*ij*1*p*+1_^(*l*)^)=*α*_*ij*1_^(*l*)^
  2. When *α*_*ij*1_^(*l*)^=0, *m*_*l*_(*S*_*ij*1*p*_^(*l*)^)=1
  3. When −0.5 < *α*_*ij*1_^(*l*)^ < 0, *m*_*l*_(*S*_*ij*1*p*−1_^(*l*)^)=|*α*_*ij*1_^(*l*)^|, *m*_*l*_(*S*_*ij*1*p*_^(*l*)^)=1 − |*α*_*ij*1_^(*l*)^|
*Step 2*. Calculate experts' reliability

**Definition 7 .**

Based on the degree of certainty of 2-dimension 2-tuple linguistic information given by experts and their experience, an expert's reliability is defined as follows:

(10) $$\begin{array}{c} \lambda^{\left(l\right)}=\frac{\lambda_{f}^{\left(l\right)}d\left(\left(S_{ij1q}^{\left(l\right)*},\alpha_{ij1}^{\left(l\right)*}\right),\left(S_{0}^{*},0\right)\right)}{\overset{h}{\underset{k=1}{\sum }}\lambda_{f}^{\left(k\right)}d\left(\left(S_{ij1q}^{\left(k\right)*},\alpha_{ij1}^{\left(k\right)*}\right),\left(S_{0}^{*},0\right)\right)},\;l=1,\ldots ,h, \end{array}$$

where *λ*_*f*_^(*l*)^ is the coefficient that reflects experts' experience and preference, 0.9 < *λ*_*f*_^(*l*)^ < 1; *λ*^(*l*)^ is called the expert reliability; *d*((*S*_*ij*1*q*_^(*l*)*∗*^, *α*^(*l*)*∗*^), (*S*_0_^*∗*^, 0)) is the distance between the second dimension linguistic information given by experts and (*S*_0_^*∗*^, 0) as defined in equation (6); and Δ^−1^ is the equivalent transformation function between 2-tuple linguistic information and the numerical value as defined in equations (2) and (3).
With respect to the different values of *S*_*q*_^(*l*)*∗*^, some special cases of *λ*^(*l*)^ are shown as follows:
  1. When (*S*_*ij*1*q*_^(*l*)*∗*^, *α*_*ij*1_^(*l*)*∗*^)=(*S*_0_^*∗*^, 0),  *λ*^(*l*)^=0
  2. When (*S*_*ij*1*q*_^(*l*)*∗*^, *α*_*ij*1_^(*l*)*∗*^) ≠ (*S*_0_^*∗*^, 0) and $\underset{w\neq \text{l,}1\leq \text{w}\leq \text{h}}{\forall }\left(S_{ij1q}^{\left(w\right)*},\alpha_{ij1}^{\left(w\right)*}\right)=\left(S_{0}^{*},0\right),\lambda^{\left(l\right)}=1$
*Step 3*. Aggregate experts' information

This paper applies evidence theory to aggregate experts' information and proposes a new evidence combination rule as follows:

(11) $$\begin{array}{c} m^{ij1}\left(S_{ij1k}\right)=\underset{S_{ij1p}^{\left(1\right)}\cap S_{ij1p}^{\left(2\right)}\cap \cdots \cap S_{ij1p}^{\left(h\right)}=S_{ij1k}}{\sum }m_{1}\left(S_{ij1p}^{\left(1\right)}\right)m_{2}\left(S_{ij1p}^{\left(2\right)}\right)\cdots m_{h}\left(S_{ij1p}^{\left(h\right)}\right)+K\overset{h}{\underset{l=1}{\sum }}\lambda^{\left(l\right)}m_{l}\left(S_{ij1k}^{\left(l\right)}\right), \end{array}$$

where *K*=∑_*S*_*ij*1*p*_^(1)^∩*S*_*ij*1*p*_^(2)^∩⋯∩*S*_*ij*1*p*_^(*h*)^=*ϕ*_*m*_1_(*S*_*ij*1*p*_^(1)^)*m*_2_(*S*_*ij*1*p*_^(2)^) ⋯ *m*_*h*_(*S*_*ij*1*p*_^(*h*)^) and *m*^*ij*1^(*S*_*ij*1*k*_) represents the basic credibility that the evaluation value of alternative AS_i_^(1)^ is *S*_*ij*1*k*_ against attribute *C*_*j*_^(1)^.

**Theorem 1 .**

Let TL_*ij*1_^(*l*)^=(*S*_*ij*1*p*_^(*l*)^, *α*_*ij*1_^(*l*)^), *i*=1,2,…, *y*,  *l*=1,2,…, *y*,  *j*=1,2,…, *n*,  *p*=0,1,…, *g*_1_ be 2-tuple linguistic information given by experts. When *h* > 2 and $\underset{l\in \left\{1,2,\ldots ,h\right\}}{\forall }\Delta^{-1}\left(S_{ij1p}^{\left(l\right)},\alpha_{ij1}^{\left(l\right)}\right)\in \left[p,p+1\right]$ or *h*=2 and Δ^−1^(*S*_*ij*1*p*_^(1)^, 0)=Δ^−1^(*S*_*ij*1*p*_^(2)^, 0), then the experts' evaluation information aggregated by evidence theory can be expressed as

(12) $$\begin{array}{c} m^{ij1}\left(S_{ij1k}\right)=\underset{S_{ij1p}^{\left(1\right)}\cap S_{ij1p}^{\left(2\right)}\cap \cdots \cap S_{ij1p}^{\left(h\right)}=S_{ij1k}}{\sum }m_{1}\left(S_{ij1p}^{\left(1\right)}\right)m_{2}\left(S_{ij1p}^{\left(2\right)}\right)\cdots m_{h}\left(S_{ij1p}^{\left(h\right)}\right), \end{array}$$

where Δ^−1^ is the equivalent transformation function between 2-tuple linguistic information and the numerical value defined in equations (2) and (3).

**Proof**

 

1. For *h* > 2, since $\underset{l\in \left\{1,2,\ldots ,h\right\}}{\forall }\Delta^{-1}\left(S_{ij1p}^{\left(l\right)},\alpha_{ij1}^{\left(l\right)}\right)\in \left[p,p+1\right]$
2. then $\underset{l\in \left\{1,2,\ldots ,h\right\}}{\forall }\;\cap \;S_{ij1p}^{\left(l\right)}\neq \phi$
3. then *K* = 0
4. thus equation (12) holds
5. For *h*=2, since Δ^−1^(*S*_*ij*1*p*_^(1)^, 0)=Δ^−1^(*S*_*ij*1*p*_^(2)^, 0)
6. then *S*_*ij*1*p*_^(1)^ ∩ *S*_*ij*1*p*_^(2)^ ≠ *ϕ*
7. then *K* = 0
8. thus equation (12) holds

When there are more than two experts and their evaluation information are aggregated between the same two standard linguistic term sets or there are two experts and their evaluation information are identical, there are no conflicts between the experts. In this case, equation (12) is equivalent to equation (7).

**Theorem 2 .**

Let TL_*ij*1_^(*l*)^=(*S*_*ij*1*p*_^(*l*)^, *α*_*ij*1_^(*l*)^),  *l*=1,2,…, *h*,  *i*=1,2,…, *y*,  *j*=1,2,…, *n*,  *p*=0,1,…, *g*_1_ be 2-tuple linguistic information given by experts. When $\underset{l\in \left\{1,2,\ldots ,h\right\}}{\forall }\alpha_{ij1}^{\left(l\right)}=0$ and *S*_*ij*1*p*_^(1)^ ≠ *S*_*ij*1*p*_^(2)^ ≠ ⋯≠*S*_*ij*1*p*_^(*h*)^, then the experts' evaluation information aggregated by evidence theory can be expressed as

(13) $$\begin{array}{c} m^{ij}\left(S_{ij1k}\right)=\overset{h}{\underset{l=1}{\sum }}\lambda^{\left(l\right)}m_{l}\left(S_{ij1k}^{\left(l\right)}\right). \end{array}$$

**Proof**

 

  When $\underset{l\in \left\{1,2,\ldots ,h\right\}}{\forall }\alpha_{ij1}^{\left(l\right)}=0$ and *S*_*ij*1*p*_^(1)^ ≠ *S*_*ij*1*p*_^(2)^ ≠ ⋯≠*S*_*ij*1*p*_^(*h*)^, since $\underset{l\in \left\{1,2,\ldots ,h\right\}}{\forall }\;\cap \;S_{ij1p}^{\left(l\right)}=\phi$
  then *K* = 1 and ∑_*S*_*ij*1*p*_^(1)^∩*S*_*ij*1*p*_^(2)^∩⋯∩*S*_*ij*1*p*_^(*h*)^=*S*_*ij*1*k*__*m*_1_(*S*_*ij*1*p*_^(1)^)*m*_2_(*S*_*ij*1*p*_^(2)^) ⋯ *m*_*h*_(*S*_*ij*1*p*_^(*h*)^)=0
  thus equation (13) holds.
  When *K* = 1, equations (7) and (8) cannot be used to aggregate the experts' evaluation information.

### 5.2. Integrate Attribute Values of Alternatives Based on Evidence Theory

On the basis of Section 5.1, the integration steps of attribute values are given as follows:

 
*Step 1*. Calculate attribute reliability
  The reliability of attributes should be related to the consistency of experts. The higher the consistency of experts, the higher the reliability of attributes. Firstly, the expert consistency coefficient is calculated, and then the expert consistency coefficient is used to measure the reliability of attributes. The distance matrix between the evaluation information of experts and the standard linguistic sets of the first dimension is established as follows:

(14) $$\begin{array}{c} D_{j}=\left[\begin{array}{cccc} d_{11} & d_{12} & \cdots & d_{1g_{1}+1}\\ d_{21} & d_{22} & \cdots & d_{2g_{1}+1}\\ \cdots & \cdots & \cdots & \cdots \\ d_{h1} & d_{h2} & \cdots & d_{hg_{1}+1} \end{array}\right], \end{array}$$

  where *d*_*lθ*_=∑_*i*=1_^*y*^*d*((*S*_*ij*1*p*_^(*l*)^, *α*_*ij*1_^(*l*)^), (*S*_*θ*−1_, 0))(*S*_*ij*1*p*_^(*l*)^, *S*_*θ*−1_ ∈ *TS*) represents the distance between the first dimension linguistic evaluation information given by experts and the standard linguistic information *S*_*θ*−1_(*S*_*θ*−1_ ∈ TS).

**Definition 8 .**

Let *γ*_*j*_=1/(min_*θ*_∑_*i*=1_^*h*^*d*_*lθ*_),  *j*=1,2,…, *n* be expert consistency coefficient; then, the attribute reliability is defined as follows:

(15) $$\begin{array}{c} \omega_{j}=\frac{\gamma_{j}}{\overset{n}{\underset{\varepsilon =1}{\sum }}\gamma_{\varepsilon }},j=1,2,...,n. \end{array}$$

  Obviously, 0 < *ω*_*j*_ < 1.
 
*Step 2*. Aggregate attribute values

According to formula (11), attribute values are integrated as

(16) $$\begin{array}{c} m^{i1}\left(S_{i1G}\right)=\underset{S_{i11k}\cap S_{i21k}\cap \cdots \cap S_{in1k}=S_{i1G}}{\sum }m^{i11}\left(S_{i11k}\right)m^{i21}\left(S_{i21k}\right)\cdots m^{in1}\left(S_{in1k}\right)+K\overset{n}{\underset{j=1}{\sum }}\omega_{j}m^{ij1}\left(S_{ij1G}\right), \end{array}$$

where *m*^*i*1^(*S*_*i*1*G*_) represents the basic credibility when the evaluation value of alternative AS_i_^(1)^ is *S*_*G*_ and *K*=∑_*S*_*i*11*k*_∩*S*_*i*21*k*_∩⋯∩*S*_*in*1*k*_=*ϕ*_  *m*^*i*11^(*S*_*i*11*k*_)*m*^*i*21^(*S*_*i*21*k*_) ⋯ *m*^*in*1^(*S*_*in*1*k*_).

### 5.3. Determine Nash Equilibrium Point

According to the discussions in Sections 5.1 and 5.2, the basic probability distribution in each alternative can be obtained. Thus, the overall values of every alternative are obtained as follows:

(17) $$\begin{array}{c} z_{i}^{\left(1\right)}=\overset{g_{1}}{\underset{G=0}{\sum }}m^{i1}\left(S_{i1G}\right)u\left(S_{i1G}\right),i=1,2,\ldots ,y. \end{array}$$

Therefore, the overall value of manufacturer *A* choosing different strategies under the condition that manufacturer *B* chooses different strategies is obtained. Under the condition that manufacturer *A* chooses different strategies, manufacturer *B* chooses the same method to determine the overall values of different strategies.

According to the overall values determined by formula (17), the game matrix is constructed as follows:

(18) $$\begin{array}{c} Z=\left[\begin{array}{cccc} z_{11}^{\left(1\right)};z_{11}^{\left(2\right)} & z_{12}^{\left(1\right)};z_{12}^{\left(2\right)} & \cdots & z_{1t}^{\left(1\right)};z_{1t}^{\left(2\right)}\\ z_{21}^{\left(1\right)};z_{21}^{\left(2\right)} & z_{22}^{\left(1\right)};z_{22}^{\left(2\right)} & \cdots & z_{2t}^{\left(1\right)};z_{2t}^{\left(2\right)}\\ \cdots & \cdots & \cdots & \cdots \\ z_{y1}^{\left(1\right)};z_{y1}^{\left(2\right)} & z_{y2}^{\left(1\right)};z_{y2}^{\left(2\right)} & \cdots & z_{yt}^{\left(1\right)};z_{yt}^{\left(2\right)} \end{array}\right], \end{array}$$

where *z*_*ij*_^(*k*)^ represents the overall value of decision maker *k* choosing strategy AS_i_^(1)^ under the condition that competitor chooses strategy AS_j_^(2)^.

According to the game matrix, the Nash equilibrium point is determined by game theory, i.e.,

(19a) $$\begin{array}{c} \left\{\begin{array}{c} \max\;z^{\left(1\right)}\left(p,q^{*}\right)=\overset{y}{\underset{i=1}{\sum }}p_{i}\left(\overset{t}{\underset{j=1}{\sum }}q_{j}^{*}z_{ij}^{\left(1\right)}\right),\\ \max\;z^{\left(2\right)}\left(p^{*},q\right)=\overset{t}{\underset{j=1}{\sum }}q_{j}\left(\overset{y}{\underset{i=1}{\sum }}p_{i}^{*}z_{ij}^{\left(2\right)}\right), \end{array}\right. \end{array}$$

(19b) $$\begin{array}{c} \text{s.t.}\left\{\begin{array}{c} \overset{y}{\underset{i=1}{\sum }}p_{i}=1\\ \begin{array}{c} \overset{t}{\underset{j=1}{\sum }}q_{j}=1\\ p_{i},q_{j}\geq 0. \end{array} \end{array}\right. \end{array}$$

By solving the objective programming models (19a) and (19b), the Nash equilibrium point (*p*^*∗*^, *q*^*∗*^) of competitive multiattribute group decision-making problem is obtained under the mixed strategy. The Nash equilibrium of mixed strategy refers to the situation that manufacturers are trying to guess each other first and trying not to let the other guess his or her strategy (e.g., mora, poker game, and game). This paper takes the case of literature [2] as the background and uses the data in the literature of [1, 21, 22, 26] to give the following illustrations.

## 6. Illustrations

Consider a duopoly market where two oligarchs produce and sell two identical products, and each oligarch's market demand curve is open to the other. Their combined output determines the market price of the product, and the oligarchs influence each other. Denote the two oligarchs as manufacturer *A* and manufacturer *B*. It is noticed that the decisions made by manufacturer *A* affect the decisions made by manufacturer *B*. In addition, the decisions made by manufacturer *A* are influenced by decisions made by manufacturer *B*.

Now, the research and development department of manufacturer *A* has discovered a new technology that can greatly improve the production efficiency of products. Faced with this situation, manufacturers *A* and *B* must reevaluate and adjust their respective market strategies. Suppose that manufacturer *A* could employ three strategies: to keep the original market strategy unchanged (AS_1_), reduce the cost with new technology and take the initiative to reduce the price (AS_2_), and reduce the unit cost with new technology but keep the original output and price unchanged (AS_3_). Manufacturer *B* can also employ three strategies as to increase R&D investment to achieve technological breakthrough as soon as possible (BS_1_), reduce the market size to maintain the market price (BS_2_), and maintain the current production scale and reduce the price (BS_3_).

The attributes for evaluations include sustainability, future development, and benefits. Each side invited three experts for evaluation and supposed that *λ*_1_={0.92, 0.93, 0.95}, *λ*_2_={0.91, 0.94, 0.95}. The standard linguistic term set of the first dimension is

(20) $$\begin{array}{c} \text{TS}=\left\{S_{0}=\text{verypoor},\;S_{1}=\text{poor},\;S_{2}=\text{littlepoor},\;S_{3}=\text{general},\;S_{4}=\text{littlegood},\;S_{5}=\text{good},\;S_{6}=\text{verygood}\right\}. \end{array}$$

The standard linguistic term set of the second dimension is

(21) $$\begin{array}{c} {\text{TS}}^{*}=\left\{S_{0}^{*}=\text{verypoor},S_{1}^{*}=\text{poor},S_{2}^{*}=\text{littlepoor},\;S_{3}^{*}=\text{general},\;S_{4}^{*}=\text{littlegood},\;S_{5}^{*}=\text{good},\;S_{6}^{*}=\text{verygood}\right\},\\ u\left(\text{TS}\right)\text{ is }\left\{0,0.1,0.3,0.6,0.7,0.9,1\right\}. \end{array}$$

Under the condition that manufacturer *B* chooses each strategy, for the strategies chosen by manufacturer *A*, experts give their evaluation information, as shown in Tables 1–3, respectively.

Under the condition that manufacturer *A* chooses each strategy, for the strategies chosen by manufacturer *B*, experts give their evaluation information, as shown in Tables 4–6, respectively.

The results obtained by aggregating the information of experts are shown in Tables 7–12. The results obtained by integrating the attribute values are shown in Tables 13–18.

The overall value of each alternative is calculated and the results are as follows.

Under the condition that manufacturer *B* chooses strategy BS_1_:
    The overall value of AS_1_ : 0.0098  *∗*  0+0.4475 *∗* 0.1 +0.5427 *∗* 0.3=0.2076
    The overall value of AS_2_ : 0.0522 *∗* 0.1+0.7606 *∗* 0.3 +0.1872 *∗* 0.6=0.3457
    The overall value of AS_3_ : 0.0097 *∗* 0.3+0.8026 *∗* 0.6 +0.1877 *∗* 0.7=0.6159
Under the condition that manufacturer *B* chooses strategy BS_2_:
    The overall value of AS_1_ : 0.0426 *∗* 0.6+0.6728 *∗* 0.7 +0.2846 *∗* 0.9=0.7527
    The overall value of AS_2_ : 0.0693 *∗* 0.6+0.758 *∗* 0.7 +0.1727 *∗* 0.9=0.7276
    The overall value of AS_3_ : 0.0408 *∗* 0.6+0.4366 *∗* 0.7 +0.5226 *∗* 0.9=0.8004
Under the condition that manufacturer B chooses strategy BS_3_:
    The overall value of AS_1_ : 0.0628 *∗* 0.6+0.7483 *∗* 0.7 +0.1889 *∗* 0.9=0.7315
    The overall value of AS_2_ : 0.0155 *∗* 0.3+0.3343 *∗* 0.6 +0.3719 *∗* 0.7+0.2559 *∗* 0.9+0.0224 *∗* 1=0.7183
    The overall value of AS_3_ : 0.013 *∗* 0.3+0.1886 *∗* 0.6 +0.2457 *∗* 0.7+0.5359 *∗* 0.9+0.0168 *∗* 1=0.7882
Under the condition that manufacturer *A* chooses strategy AS_1_:
    The overall value of BS_1_ : 0.0512 *∗* 0.3+0.3258 *∗* 0.6 +0.3722 *∗* 0.7+0.2508 *∗* 0.9=0.6971
    The overall value of BS_2_ : 0.0306 *∗* 0.6+0.9461 *∗* 0.7 +0.0233 *∗* 0.9=0.7016
    The overall value of BS_3_ : 0.0576 *∗* 0.3+0.579 *∗* 0.6 +0.2504 *∗* 0.7+0.113 *∗* 0.9=0.6417
Under the condition that manufacturer *A* chooses strategy AS_2_:
    The overall value of BS_1_ : 0.3799 *∗* 0.6+0.4846 *∗* 0.7 +0.1355 *∗* 0.9=0.6891
    The overall value of BS_2_ : 0.0128 *∗* 0.3+0.2355 *∗* 0.6 +0.7082 *∗* 0.7+0.0435 *∗* 0.9=0.68
    The overall value of BS_3_ : 0.5692 *∗* 0.6+0.3044 *∗* 0.7 +0.1264 *∗* 0.9=0.6684
Under the condition that manufacturer *A* chooses strategy AS_3_:
    The overall value of BS_1_ : 0.0169 *∗* 0.3+0.1364 *∗* 0.6 +0.6699 *∗* 0.7+0.1768 *∗* 0.9=0.715
    The overall value of BS_2_ : 0.3417 *∗* 0.3+0.5953 *∗* 0.6 +0.2974 *∗* 0.7+0.0045 *∗* 0.9=0.5525
    The overall value of BS_3_ : 0.1887 *∗* 0.6+0.6227 *∗* 0.7 +0.1886 *∗* 0.9=0.7188

According to the above results, the bivariate decision matrix is constructed as follows:

(22) $$\begin{array}{c} Z=\left(\begin{array}{ccc} 0.2076;0.6971 & 0.7527;0.6891 & 0.7315;0.715\\ 0.3457;0.7016 & 0.7276;0.68 & 0.7182;0.5525\\ 0.6159;0.6417 & 0.8004;0.6684 & 0.7882;0.7188 \end{array}\right). \end{array}$$

Obviously, there is a pure strategy Nash equilibrium in this case (AS_3_, BS_3_). The corresponding revenue is (0.7882, 0.7188).

## 7. Conclusions

In this paper, based on game theory and evidence theory, an approach is proposed for the competitive multiattribute group decision-making problem of the game between manufacturers, with the attribute values being the 2-dimension 2-tuple linguistic evaluation information. Firstly, in order to aggregate experts' evaluation information, a method is proposed to determine the 2-dimension 2-tuple linguistic basic probability distribution function, and the experts' reliability is defined. After that, the evidence theory is applied again to conduct secondary aggregation with attribute values as the evidence source, and the consistency degree of experts is taken as the criterion to measure the reliability of attribute evidence source. Finally, the Nash equilibrium point of competitive multiattribute group decision-making problem is determined by game theory.

Compared with the traditional competitive multiattribute group decision-making problem, the 2-dimension 2-tuple linguistic competitive multiattribute group decision-making problem has higher certainty and accuracy, which makes the decision results more reliable and have very important significance.

In addition, in the course of aggregating experts' evaluation information, the proposed approach could solve the conflict of evidence, that is, from the subjective dimension and the objective dimension, to determine the reliability of experts' evidence. The subjective dimension uses background and authority of experts [30], while the objective dimension uses the second semantic information of the degree of certainty, which overcomes the problem of too much subjectivity when the original evidence theory solves conflicts.

## Figures and Tables

**Table 1:** Experts' evaluation information of every strategy for manufacturer *A* under the condition that manufacturer *B* chooses strategy BS_1_.

	Expert 1			Expert 2			Expert 3		
	*C*_1_	*C*_2_	*C*_3_	*C*_1_	*C*_2_	*C*_3_	*C*_1_	*C*_2_	*C*_3_
AS_1_	(*S*_2_, −0.2), (*S*_3_^*∗*^, 0.3)	(*S*_1_, −0.05), (*S*_3_^*∗*^, 0.2)	(*S*_2_, −0.4), (*S*_3_^*∗*^, 0)	(*S*_1_, −0.1), (*S*_3_^*∗*^, −0.15)	(*S*_2_, −0.2), (*S*_3_^*∗*^, 0.35)	(*S*_1_, 0.35), (*S*_2_^*∗*^, 0.4)	(*S*_2_, −0.15), (*S*_3_^*∗*^, −0.3)	(*S*_1_, 0.1), (*S*_3_^*∗*^, −0.5)	(*S*_2_, 0), (*S*_3_^*∗*^, −0.35)
AS_2_	(*S*_2_, 0), (*S*_3_^*∗*^, −0.45)	(*S*_1_, 0), (*S*_3_^*∗*^, 0.35)	(*S*_2_, 0), (*S*_3_^*∗*^, 0.05)	(*S*_3_, −0.4), (*S*_2_^*∗*^, 0.398)	(*S*_2_, 0.24), (*S*_2_^*∗*^, 0.484)	(*S*_3_, −0.4), (*S*_2_^*∗*^, 0.484)	(*S*_2_, 0.12), (*S*_4_^*∗*^, 0)	(*S*_2_, 0.24), (*S*_4_^*∗*^, 0)	(*S*_3_, −0.24), (*S*_4_^*∗*^, 0)
AS_3_	(*S*_2_, 0.36), (*S*_4_^*∗*^, 0)	(*S*_3_, −0.16), (*S*_4_^*∗*^, 0)	(*S*_3_, −0.16), (*S*_4_^*∗*^, 0)	(*S*_3_, 0.6), (*S*_4_^*∗*^, 0)	(*S*_3_, 0.32), (*S*_3_^*∗*^, −0.214)	(*S*_3_, 0.24), (*S*_3_^*∗*^, −0.214)	(*S*_3_, 0.08), (*S*_2_^*∗*^, −0.111)	(*S*_4_, −0.4), (*S*_2_^*∗*^, −0.111)	(*S*_4_, −0.32), (*S*_2_^*∗*^, −0.111)

**Table 2:** Experts' evaluation information of every strategy for manufacturer *A* under the condition that manufacturer *B* chooses strategy BS_2_.

	Expert 1			Expert 2			Expert 3		
	*C*_1_	*C*_2_	*C*_3_	*C*_1_	*C*_2_	*C*_3_	*C*_1_	*C*_2_	*C*_3_
AS_1_	(*S*_5_, −0.32), (*S*_2_^*∗*^, 0.398)	(*S*_5_, −0.32), (*S*_4_^*∗*^, 0)	(*S*_4_, −0.4), (*S*_2_^*∗*^, 0.398)	(*S*_4_, 0.32), (*S*_4_^*∗*^, 0)	(*S*_4_, 0.16), (*S*_2_^*∗*^, 0.398)	(*S*_5_, −0.16), (*S*_4_^*∗*^, 0)	(*S*_4,_ − 0.24), (*S*_2_^*∗*^, 0.484)	(*S*_4_, 0.36), (*S*_3_^*∗*^, −0.214)	(*S*_4_, −0.12), (*S*_2_^*∗*^, −0.484)
AS_2_	(*S*_4_, 0.28), (*S*_3_^*∗*^, −0.214)	(*S*_4_, −0.32), (*S*_4_^*∗*^, 0)	(*S*_4_, 0.4), (*S*_4_^*∗*^, 0)	(*S*_4_, −0.08), (*S*_2_^*∗*^, −0.111)	(*S*_5_, −0.08), (*S*_4_^*∗*^, 0)	(*S*_3_, 0.4), (*S*_4_^*∗*^, 0)	(*S*_4_, 0.4), (*S*_4_^*∗*^, 0)	(*S*_4_, 0.28), (*S*_4_^*∗*^, 0)	(*S*_4_, −0.21), (*S*_3_^*∗*^, 0)
AS_3_	(*S*_4_, 0.21), (*S*_3_^*∗*^, 0)	(*S*_5_, −0.19), (*S*_3_^*∗*^, 0)	(*S*_5_, −0.19), (*S*_3_^*∗*^, 0)	(*S*_4_, −0.4), (*S*_3_^*∗*^, 0)	(*S*_4_, 0.01), (*S*_3_^*∗*^, 0)	(*S*_5_, −0.19), (*S*_3_^*∗*^, 0)	(*S*_5_, −0.19), (*S*_3_^*∗*^, 0)	(*S*_4_, 0.4), (*S*_3_^*∗*^, 0)	(*S*_5_, −0.2), (*S*_3_^*∗*^, 0)

**Table 3:** Experts' evaluation information of every strategy for manufacturer *A* under the condition that manufacturer *B* chooses strategy BS_3_.

	Expert 1			Expert 2			Expert 3		
	*C*_1_	*C*_2_	*C*_3_	*C*_1_	*C*_2_	*C*_3_	*C*_1_	*C*_2_	*C*_3_
AS_1_	(*S*_3_, 0.21), (*S*_2_^*∗*^, 0)	(*S*_4_, 0), (*S*_3_^*∗*^, 0)	(*S*_4_, 0), (*S*_2_^*∗*^, 0)	(*S*_4_, 0.4), (*S*_2_^*∗*^, 0)	(*S*_4_, −0.19), (*S*_2_^*∗*^, 0)	(*S*_4_, 0.2), (*S*_3_^*∗*^, 0)	(*S*_4_, 0.19), (*S*_2_^*∗*^, 0)	(*S*_5_, −0.2), (*S*_3_^*∗*^, 0)	(*S*_5_, −0.2), (*S*_3_^*∗*^, 0)
AS_2_	(*S*_3_, −0.2), (*S*_2_^*∗*^, 0)	(*S*_3_, 0.2), (*S*_2_^*∗*^, 0)	(*S*_3_, −0.01), (*S*_2_^*∗*^, 0)	(*S*_3_, 0.19), (*S*_3_^*∗*^, 0)	(*S*_4_, 0.4), (*S*_2_^*∗*^, 0)	(*S*_5_, 0.2), (*S*_3_^*∗*^, 0)	(*S*_4_, 0), (*S*_3_^*∗*^, 0)	(*S*_4_, 0.4), (*S*_3_^*∗*^, 0)	(*S*_5_, −0.2), (*S*_2_^*∗*^, 0)
AS_3_	(*S*_5_, 0.01), (*S*_3_^*∗*^, 0)	(*S*_5_, −0.19), (*S*_3_^*∗*^, 0)	(*S*_5_, 0.2), (*S*_3_^*∗*^, 0)	(*S*_3_, −0.19), (*S*_3_^*∗*^, 0)	(*S*_3_, 0.4), (*S*_2_^*∗*^, 0)	(*S*_3_, 0.01), (*S*_2_^*∗*^, 0)	(*S*_4_, −0.19), (*S*_3_^*∗*^, 0)	(*S*_4_, 0.39), (*S*_3_^*∗*^, 0)	(*S*_5_, −0.02), (*S*_4_^*∗*^, 0)

**Table 4:** Experts' evaluation information of every strategy for manufacturer *B* under the condition that manufacturer *A* chooses strategy AS_1_.

	Expert 1			Expert 2			Expert 3		
	*C*_1_	*C*_2_	*C*_3_	*C*_1_	*C*_2_	*C*_3_	*C*_1_	*C*_2_	*C*_3_
BS_1_	(*S*_3_, −0.4), (*S*_2_^*∗*^, 0)	(*S*_3_, 0.4), (*S*_3_^*∗*^, 0)	(*S*_3_, −0.19), (*S*_2_^*∗*^, 0)	(*S*_4_, −0.39), (*S*_3_^*∗*^, 0)	(*S*_4_, 0.3182), (*S*_3_^*∗*^, 0)	(*S*_5_, −0.4142), (*S*_2_^*∗*^, 0)	(*S*_5_, −0.1892), (*S*_2_^*∗*^, 0)	(*S*_5_, 0), (*S*_2_^*∗*^, 0)	(*S*_4_, −0.4495), (*S*_3_^*∗*^, 0)
BS_2_	(*S*_4_, −0.2795), (*S*_4_^*∗*^, 0)	(*S*_4_, −0.0598), (*S*_1_^*∗*^, 0)	(*S*_4_, 0), (*S*_2_^*∗*^, 0)	(*S*_4_, 0.1388), (*S*_2_^*∗*^, 0)	(*S*_4_, 0.3182), (*S*_1_^*∗*^, 0)	(*S*_4_, −0.2795), (*S*_4_^*∗*^, 0)	(*S*_4_, 0.1388), (*S*_3_^*∗*^, 0)	(*S*_4_, −0.3784), (*S*_4_^*∗*^, 0)	(*S*_4_, −0.2134), (*S*_4_^*∗*^, 0)
BS_3_	(*S*_4_, 0), (*S*_2_^*∗*^, 0)	(*S*_4_, 0.3182), (*S*_3_^*∗*^, 0)	(*S*_4_, 0.4349), (*S*_3_^*∗*^, 0)	(*S*_5_, −0.4142), (*S*_2_^*∗*^, 0)	(*S*_3_, −0.3437), (*S*_4_^*∗*^, 0)	(*S*_3_, −0.1623), (*S*_3_^*∗*^, 0)	(*S*_3_, 0.0093), (*S*_3_^*∗*^, 0)	(*S*_3_, 0.1716), (*S*_3_^*∗*^, 0)	(*S*_3_, 0.0093), (*S*_3_^*∗*^, 0)

**Table 5:** Experts' evaluation information of every strategy for manufacturer *B* under the condition that manufacturer *A* chooses strategy AS_2_.

	Expert 1			Expert 2			Expert 3		
	*C*_1_	*C*_2_	*C*_3_	*C*_1_	*C*_2_	*C*_3_	*C*_1_	*C*_2_	*C*_3_
BS_1_	(*S*_3_, 0.1716), (*S*_4_^*∗*^, 0)	(*S*_3_, 0.41), (*S*_3_^*∗*^, 0)	(*S*_4_, −0.4495), (*S*_4_^*∗*^, 0)	(*S*_4_, 0.2679), (*S*_3_^*∗*^, 0)	(*S*_4_, 0.4349), (*S*_3_^*∗*^, 0)	(*S*_4_, 0.4349), (*S*_3_^*∗*^, 0)	(*S*_4_, 0.4142), (*S*_3_^*∗*^, 0)	(*S*_3_, 0.087), (*S*_4_^*∗*^, 0)	(*S*_3_, 0.2892), (*S*_1_^*∗*^, 0)
BS_2_	(*S*_4_, −0.4495), (*S*_5_^*∗*^, 0)	(*S*_4_, −0.3784), (*S*_4_^*∗*^, 0)	(*S*_4_, −0.2795), (*S*_1_^*∗*^, 0)	(*S*_4_, −0.2134), (*S*_1_^*∗*^, 0)	(*S*_4_, 0), (*S*_4_^*∗*^, 0)	(*S*_4_, 0.4349), (*S*_1_^*∗*^, 0)	(*S*_4_, 0.3182), (*S*_3_^*∗*^, 0)	(*S*_4_, 0.1388), (*S*_1_^*∗*^, 0)	(*S*_3_, −0.0801), (*S*_4_^*∗*^, 0)
BS_3_	(*S*_3_, 0.087), (*S*_4_^*∗*^, 0)	(*S*_3_, 0.2892), (*S*_2_^*∗*^, 0)	(*S*_3_, 0.4851), (*S*_3_^*∗*^, 0)	(*S*_4_, −0.3784), (*S*_3_^*∗*^, 0)	(*S*_4_, −0.2134), (*S*_2_^*∗*^, 0)	(*S*_2_, 0.064), (*S*_4_^*∗*^, 0)	(*S*_2_, 0.2776), (*S*_3_^*∗*^, 0)	(*S*_3_, 0.2892), (*S*_1_^*∗*^, 0)	(*S*_4_, −0.2795), (*S*_2_^*∗*^, 0)

**Table 6:** Experts' evaluation information of every strategy for manufacturer *B* under the condition that manufacturer *A* chooses strategy AS_3_.

	Expert 1			Expert 2			Expert 3		
	*C*_1_	*C*_2_	*C*_3_	*C*_1_	*C*_2_	*C*_3_	*C*_1_	*C*_2_	*C*_3_
BS_1_	(*S*_4_, 0.1388), (*S*_3_^*∗*^, 0)	(*S*_4_, 0.1388), (*S*_4_^*∗*^, 0)	(*S*_4_, 0.2217), (*S*_3_^*∗*^, 0)	(*S*_4_, 0.4349), (*S*_3_^*∗*^, 0)	(*S*_5_, −0.4953), (*S*_3_^*∗*^, 0)	(*S*_5_, −0.4142), (*S*_3_^*∗*^, 0)	(*S*_5_, −0.1892), (*S*_3_^*∗*^, 0)	(*S*_3_, 0.3679), (*S*_2_^*∗*^, 0)	(*S*_3_, 0.4851), (*S*_2_^*∗*^, 0)
BS_2_	(*S*_3_, 0.087), (*S*_2_^*∗*^, 0)	(*S*_3_, 0.2168), (*S*_2_^*∗*^, 0)	(*S*_3_, −0.2237), (*S*_2_^*∗*^, 0)	(*S*_3_, −0.0801), (*S*_2_^*∗*^, 0)	(*S*_2_, 0.4324), (*S*_4_^*∗*^, 0)	(*S*_3_, −0.4087), (*S*_2_^*∗*^, 0)	(*S*_3_, −0.4087), (*S*_2_^*∗*^, 0)	(*S*_3_, −0.2237), (*S*_3_^*∗*^, 0)	(*S*_3_, 0), (*S*_4_^*∗*^, 0)
BS_3_	(*S*_4_, 0.2217), (*S*_3_^*∗*^, 0)	(*S*_4_, 0.3182), (*S*_2_^*∗*^, 0)	(*S*_4_, 0.2217), (*S*_3_^*∗*^, 0)	(*S*_3_, 0.3182), (*S*_3_^*∗*^, 0)	(*S*_4_, 0.4349), (*S*_2_^*∗*^, 0)	(*S*_5_, −0.4953), (*S*_3_^*∗*^, 0)	(*S*_5_, −0.4142), (*S*_3_^*∗*^, 0)	(*S*_5_, −0.3161), (*S*_2_^*∗*^, 0)	(*S*_5_, −0.3161), (*S*_2_^*∗*^, 0)

**Table 7:** The aggregation results of experts' evaluation information for manufacturer *A* under the condition that manufacturer *B* chooses strategy BS_1_.

	*C*_1_	*C*_2_	*C*_3_
AS_1_	*m*(*S*_0_)=0.0312, *m*(*S*_1_)=0.4253, *m*(*S*_2_)=0.5435	*m*(*S*_1_)=0.7146, *m*(*S*_2_)=0.2854	*m*(*S*_1_)=0.2688, *m*(*S*_2_)=0.7312
AS_2_	*m*(*S*_2_)=0.8612, *m*(*S*_3_)=0.1388	*m*(*S*_1_)=0.2614, *m*(*S*_2_)=0.5614 *m*(*S*_3_)=0.1772	*m*(*S*_2_)=0.5667, *m*(*S*_3_)=0.4333
AS_3_	*m*(*S*_3_)=0.682, *m*(*S*_4_)=0.318	*m*(*S*_2_)=0.0563, *m*(*S*_3_)=0.7614 *m*(*S*_4_)=0.1823	*m*(*S*_3_)=0.6986, *m*(*S*_4_)=0.3014

**Table 8:** The aggregation results of experts' evaluation information for manufacturer *A* under the condition that manufacturer *B* chooses strategy BS_2_.

	*C*_1_	*C*_2_	*C*_3_
AS_1_	*m*(*S*_3_)=0.057, *m*(*S*_4_)=0.6719, *m*(*S*_5_)=0.272	*m*(*S*_4_)=0.6094, *m*(*S*_5_)=0.3906	*m*(*S*_3_)=0.1288, *m*(*S*_4_)=0.5259 *m*(*S*_5_)=0.3453
AS_2_	*m*(*S*_3_)=0.0104, *m*(*S*_4_)=0.8235 *m*(*S*_5_)=0.1661	*m*(*S*_3_)=0.0955, *m*(*S*_4_)=0.5092 *m*(*S*_5_)=0.3953	*m*(*S*_3_)=0.2238, *m*(*S*_4_)=0.6598 *m*(*S*_5_)=0.1164
AS_3_	*m*(*S*_3_)=0.1209, *m*(*S*_4_)=0.5663, *m*(*S*_5_)=0.3128	*m*(*S*_4_)=0.6386, *m*(*S*_5_)=0.3614	*m*(*S*_4_)=0.0977, *m*(*S*_5_)=0.9023

**Table 9:** The aggregation results of experts' evaluation information for manufacturer *A* under the condition that manufacturer *B* chooses strategy BS_3_.

	*C*_1_	*C*_2_	*C*_3_
AS_1_	*m*(*S*_3_)=0.2331, *m*(*S*_4_)=0.5897, *m*(*S*_5_)=0.1772	*m*(*S*_3_)=0.0396, *m*(*S*_4_)=0.7046 *m*(*S*_5_)=0.2558	*m*(*S*_4_)=0.6813, *m*(*S*_5_)=0.3187
AS_2_	*m*(*S*_2_)=0.0492, *m*(*S*_3_)=0.4989, *m*(*S*_4_)=0.4519	*m*(*S*_3_)=0.2086, *m*(*S*_4_)=0.5245 *m*(*S*_5_)=0.2669	*m*(*S*_2_)=0.0028, *m*(*S*_3_)=0.279 *m*(*S*_4_)=0.5163, *m*(*S*_5_)=0.1164 *m*(*S*_6_)=0.0855
AS_3_	*m*(*S*_3_)=0.2742, *m*(*S*_4_)=0.309, *m*(*S*_5_)=0.3658 *m*(*S*_2_)=0.0473, *m*(*S*_6_)=0.0037	*m*(*S*_4_)=0.4302, *m*(*S*_5_)=0.4273 *m*(*S*_3_)=0.1425	*m*(*S*_4_)=0.0112, *m*(*S*_5_)=0.7045 *m*(*S*_3_)=0.2187, *m*(*S*_6_)=0.0656

**Table 10:** The aggregation results of experts' evaluation information for manufacturer *B* under the condition that manufacturer *A* chooses strategy AS_1_.

	*C*_1_	*C*_2_	*C*_3_
BS_1_	*m*(*S*_3_)=0.3351, *m*(*S*_4_)=0.318, *m*(*S*_5_)=0.2356 *m*(*S*_2_)=0.1113	*m*(*S*_3_)=0.2199, *m*(*S*_4_)=0.4046 *m*(*S*_5_)=0.3755	*m*(*S*_4_)=0.3584, *m*(*S*_5_)=0.1681 *m*(*S*_2_)=0.0528, *m*(*S*_3_)=0.4207
BS_2_	*m*(*S*_5_)=0.0365, *m*(*S*_3_)=0.0566, *m*(*S*_4_)=0.9069	*m*(*S*_3_)=0.1589, *m*(*S*_4_)=0.8093 *m*(*S*_5_)=0.0318	*m*(*S*_4_)=0.914, *m*(*S*_3_)=0.086
BS_3_	*m*(*S*_3_)=0.4294, *m*(*S*_4_)=0.4031, *m*(*S*_5_)=0.1675	*m*(*S*_4_)=0.2286, *m*(*S*_5_)=0.0845 *m*(*S*_3_)=0.5297, *m*(*S*_2_)=0.1572	*m*(*S*_4_)=0.1868, *m*(*S*_5_)=0.1413 *m*(*S*_3_)=0.6174, *m*(*S*_2_)=0.0545

**Table 11:** The aggregation results of experts' evaluation information for manufacturer *B* under the condition that manufacturer *A* chooses strategy AS_2_.

	*C*_1_	*C*_2_	*C*_3_
BS_1_	*m*(*S*_3_)=0.3, *m*(*S*_4_)=0.5074, *m*(*S*_5_)=0.1926	*m*(*S*_3_)=0.5324, *m*(*S*_4_)=0.3391 *m*(*S*_5_)=0.1285	*m*(*S*_4_)=0.5655, *m*(*S*_5_)=0.1506 *m*(*S*_3_)=0.2839
BS_2_	*m*(*S*_5_)=0.0913, *m*(*S*_3_)=0.1677, *m*(*S*_4_)=0.7409	*m*(*S*_3_)=0.0767, *m*(*S*_4_)=0.916 *m*(*S*_5_)=0.0073	*m*(*S*_4_)=0.2101, *m*(*S*_3_)=0.6637 *m*(*S*_2_)=0.0538, *m*(*S*_5_)=0.0724
BS_3_	*m*(*S*_3_)=0.5986, *m*(*S*_4_)=0.2011, *m*(*S*_5_)=0.2003	*m*(*S*_4_)=0.471, *m*(*S*_3_)=0.529	*m*(*S*_4_)=0.2663, *m*(*S*_5_)=0.3181 *m*(*S*_3_)=0.4156

**Table 12:** The aggregation results of experts' evaluation information for manufacturer *B* under the condition that manufacturer *A* chooses strategy AS_3_.

	*C*_1_	*C*_2_	*C*_3_
BS_1_	*m*(*S*_2_)=0.0642, *m*(*S*_3_)=0.2751, *m*(*S*_4_)=0.4696 *m*(*S*_5_)=0.1911	*m*(*S*_3_)=0.1211, *m*(*S*_4_)=0.6844 *m*(*S*_5_)=0.1945	*m*(*S*_4_)=0.6336, *m*(*S*_5_)=0.2556 *m*(*S*_3_)=0.1108
BS_2_	*m*(*S*_5_)=0.0142, *m*(*S*_3_)=0.0833, *m*(*S*_4_)=0.9025	*m*(*S*_3_)=0.7231, *m*(*S*_4_)=0.0345 *m*(*S*_2_)=0.2424	*m*(*S*_2_)=0.0848, *m*(*S*_3_)=0.9152
BS_3_	*m*(*S*_3_)=0.2054, *m*(*S*_4_)=0.5516, *m*(*S*_5_)=0.243	*m*(*S*_4_)=0.5281, *m*(*S*_3_)=0.4719	*m*(*S*_4_)=0.5654, *m*(*S*_5_)=0.4346

**Table 13:** The integration results of the attribute values for manufacturer *A* under the condition that manufacturer *B* chooses strategy BS_1_.

Strategies	The integration results of the attribute values
AS_1_	*m*(*S*_0_)=0.0098, *m*(*S*_1_)=0.4475, *m*(*S*_2_)=0.5427
AS_2_	*m*(*S*_1_)=0.0522, *m*(*S*_2_)=0.7606, *m*(*S*_3_)=0.1872
AS_3_	*m*(*S*_2_)=0.0097, *m*(*S*_3_)=0.8026, *m*(*S*_4_)=0.1877

**Table 14:** The integration results of the attribute values for manufacturer *A* under the condition that manufacturer *B* chooses strategy BS_2_.

Strategies	The integration results of the attribute values
AS_1_	*m*(*S*_3_)=0.0426, *m*(*S*_4_)=0.6728, *m*(*S*_5_)=0.2846
AS_2_	*m*(*S*_3_)=0.0693, *m*(*S*_4_)=0.758, *m*(*S*_5_)=0.1727
AS_3_	*m*(*S*_3_)=0.0408, *m*(*S*_4_)=0.4366, *m*(*S*_5_)=0.5226

**Table 15:** The integration results of the attribute values for manufacturer *A* under the condition that manufacturer *B* chooses strategy BS_3_.

Strategies	The integration results of the attribute values
AS_1_	*m*(*S*_3_)=0.0628, *m*(*S*_4_)=0.7483, *m*(*S*_5_)=0.1889
AS_2_	*m*(*S*_2_)=0.0155, *m*(*S*_3_)=0.3343, *m*(*S*_4_)=0.3719, *m*(*S*_5_)=0.2559, *m*(*S*_6_)=0.0224
AS_3_	*m*(*S*_2_)=0.013, *m*(*S*_3_)=0.1886, *m*(*S*_4_)=0.2457, *m*(*S*_5_)=0.5359, *m*(*S*_6_)=0.0168

**Table 16:** The integration results of the attribute values for manufacturer *B* under the condition that manufacturer *A* chooses strategy AS_1_.

Strategies	The integration results of the attribute values
BS_1_	*m*(*S*_2_)=0.0512, *m*(*S*_3_)=0.3258, *m*(*S*_4_)=0.3722, *m*(*S*_5_)=0.2508
BS_2_	*m*(*S*_3_)=0.0306, *m*(*S*_4_)=0.9461, *m*(*S*_5_)=0.0233
BS_3_	*m*(*S*_2_)=0.0576, *m*(*S*_3_)=0.579, *m*(*S*_4_)=0.2504, *m*(*S*_5_)=0.113

**Table 17:** The integration results of the attribute values for manufacturer *B* under the condition that manufacturer *A* chooses strategy AS_2_.

Strategies	The integration results of the attribute values
BS_1_	*m*(*S*_3_)=0.3799, *m*(*S*_4_)=0.4846, *m*(*S*_5_)=0.1355
BS_2_	*m*(*S*_2_)=0.0128, *m*(*S*_3_)=0.2355, *m*(*S*_4_)=0.7082, *m*(*S*_5_)=0.0435
BS_3_	*m*(*S*_3_)=0.5692, *m*(*S*_4_)=0.3044, *m*(*S*_5_)=0.1264

**Table 18:** The integration results of the attribute values for manufacturer *B* under the condition that manufacturer *A* chooses strategy AS_3_.

Strategies	The integration results of the attribute values
BS_1_	*m*(*S*_2_)=0.0169, *m*(*S*_3_)=0.1364, *m*(*S*_4_)=0.6699, *m*(*S*_5_)=0.1768
BS_2_	*m*(*S*_2_)=0.2223, *m*(*S*_3_)=0.5953, *m*(*S*_4_)=0.1779, *m*(*S*_5_)=0.0045
BS_3_	*m*(*S*_3_)=0.1887, *m*(*S*_4_)=0.6227, *m*(*S*_5_)=0.1886

## Data Availability

The data used to support the findings of this study are available from the corresponding author upon request.

## References

[1] Liu X., Ju Y., Qu Q. (2018). Hesitant fuzzy 2-dimension linguistic term set and its application to multiple attribute group decision making. *International Journal of Fuzzy Systems*.

[2] Deng X., Zheng X., Su X. (2014). An evidential game theory framework in multi-criteria decision making process. *Applied Mathematics and Computation*.

[3] Xu Z. (2007). Multi-person multi-attribute decision making models under intuitionistic fuzzy environment. *Fuzzy Optimization and Decision Making*.

[4] Li D.-F. (2007). A fuzzy closeness approach to fuzzy multi-attribute decision making. *Fuzzy Optimization and Decision Making*.

[5] Xu X., Cai X., Zhou Y. (2015). A multi-attribute large group emergency decision making method based on group preference consistency of generalized interval-valued trapezoidal fuzzy numbers. *Journal of Systems Science and Systems Engineering*.

[6] Wu Q., Lin W., Zhou L., Chen Y., Chen H. (2019). Enhancing multiple attribute group decision making flexibility based on information fusion technique and hesitant Pythagorean fuzzy sets. *Computers & Industrial Engineering*.

[7] Xiao P., Wu Q., Li H., Zhou L., Tao Z., Liu J. (2019). Novel hesitant fuzzy linguistic multi-attribute group decision making method based on improved supplementary regulation and operational laws. *IEEE Access*.

[8] Pang Q., Wang H., Xu Z. (2016). Probabilistic linguistic term sets in multi-attribute group decision making. *Information Sciences*.

[9] Li D.-F. (2009). Multiattribute group decision making method using extended linguistic variables. *International Journal of Uncertainty, Fuzziness and Knowledge-Based Systems*.

[10] Meng F., Tang J. (2014). Extended 2-tuple linguistic hybrid aggregation operators and their application to multi-attribute group decision making. *International Journal of Computational Intelligence Systems*.

[11] Wu Q., Wu P., Zhou Y., Zhou L., Chen H., Ma X. (2015). Some 2-tuple linguistic generalized power aggregation operators and their applications to multiple attribute group decision making. *Journal of Intelligent & Fuzzy Systems*.

[12] Wu Q., Zhou L., Chen Y., Chen H. (2019). An integrated approach to green supplier selection based on the interval type-2 fuzzy best-worst and extended VIKOR methods. *Information Sciences*.

[13] Herrera F., Martínez L. (1999). A 2-tuple fuzzy linguistic representation model for computing with words. *IEEE Transactions on Fuzzy Systems*.

[14] Zhu W. D., Zhou G. Z., Yang S. L. (2009). An approach to group decision making based on 2-dimension linguistic assessment information. *Systems Engineering*.

[15] Rahman K., Abdullah S., Jamil M., Khan M. Y. (2018). Some generalized intuitionistic fuzzy einstein hybrid aggregation operators and their application to multiple attribute group decision making. *International Journal of Fuzzy Systems*.

[16] Garg H., Nancy (2018). Linguistic single-valued neutrosophic prioritized aggregation operators and their applications to multiple-attribute group decision-making. *Journal of Ambient Intelligence and Humanized Computing*.

[17] Verma R. (2016). Multiple attribute group decision making based on generalized trapezoid fuzzy linguistic prioritized weighted average operator. *International Journal of Machine Learning & Cybernetics*.

[18] Xu Z., Cai X. (2012). Minimizing group discordance optimization model for deriving expert weights. *Group Decision and Negotiation*.

[19] Abootalebi S., Hadi-Vencheh A., Jamshidi A. (2018). An improvement to determining expert weights in group multiple attribute decision making problem. *Group Decision and Negotiation*.

[20] Lin M., Xu Z., Zhai Y., Yao Z. (2018). Multi-attribute group decision-making under probabilistic uncertain linguistic environment. *Journal of the Operational Research Society*.

[21] Liu P., Yu X. (2014). 2-Dimension uncertain linguistic power generalized weighted aggregation operator and its application in multiple attribute group decision making. *Knowledge-Based Systems*.

[22] Zhu H., Zhao J., Xu Y. (2016). 2-dimension linguistic computational model with 2-tuples for multi-attribute group decision making. *Knowledge-Based Systems*.

[23] Liu P., Qi X. (2014). Some generalized dependent aggregation operators with 2-dimension linguistic information and their application to group decision making. *Journal of Intelligent and Fuzzy Systems*.

[24] Yu X., Xu Z., Liu S., Chen Q. (2012). Multicriteria decision making with 2-dimension linguistic aggregation techniques. *International Journal of Intelligent Systems*.

[25] Liu P., Teng F. (2018). Multiple attribute decision-making method based on 2-dimension uncertain linguistic density generalized hybrid weighted averaging operator. *Soft Computing*.

[26] Liu P., Wang Y. (2015). The aggregation operators based on the 2-dimension uncertain linguistic information and their application to decision making. *International Journal of Machine Learning & Cybernetics*.

[27] Wu Q., Wang F., Zhou L., Chen H. (2017). Method of multiple attribute group decision making based on 2-dimension interval type-2 fuzzy aggregation operators with multi-granularity linguistic information. *International Journal of Fuzzy Systems*.

[28] Hua Z., Gong B., Xu X. (2008). A DS-AHP approach for multi-attribute decision making problem with incomplete information. *Expert Systems with Applications*.

[29] Gong B., Hua Z. The evidential reasoning approach for multi-attribute decision making problem with incomplete decision matrix.

[30] Merigó J. M., Casanovas M., Martínez L. (2010). Linguistic aggregation operators for linguistic decision making based on the DEMPSTER-SHAFER theory of evidence. *International Journal of Uncertainty, Fuzziness and Knowledge-Based Systems*.

[31] Chen Y.-W., Larbani M. (2006). Two-person zero-sum game approach for fuzzy multiple attribute decision making problems. *Fuzzy Sets and Systems*.

[32] Zhou L., Liu W., Xu Y. A game method for multiple attribute decision-making without weight information.

[33] Wei G. W. (2008). Two-tuple linguistic multiple attribute group decision making with incomplete attribute weight information. *Journal of Systems Engineering & Electronics*.

